# A High-Quality Underwater Acoustic Dataset for Algorithm Development and Analysis

**DOI:** 10.1038/s41597-025-05564-x

**Published:** 2025-07-30

**Authors:** Victor Lobo, Nuno Pessanha Santos, Ricardo Moura

**Affiliations:** 1 Portuguese Navy Research Center (CINAV), Portuguese Naval Academy (Escola Naval), Almada, 2810-001 Portugal; 2 NOVA Information Management School (Nova IMS), Universidade Nova de Lisboa, Lisbon, 1070-312 Portugal; 3 Portuguese Military Research Center (CINAMIL), Portuguese Military Academy (Academia Militar), Lisbon, 1169-203 Portugal; 4 Institute for Systems and Robotics (ISR), Instituto Superior Técnico (IST), Lisbon, 1049-001 Portugal; 5 Centro de Matemática e Aplicações (NovaMath), Universidade Nova de Lisboa, 2829-516 Caparica, Portugal

**Keywords:** Ocean sciences, Engineering

## Abstract

As data becomes increasingly available, relying on quality datasets for algorithm analysis and development is essential. However, data gathering can be expensive and time-consuming, and this process must be optimized to allow others to reuse data with simplicity and accuracy. The Wolfset is an acoustic dataset gathered using a Bruel & Kjaer type 8104 hydrophone in an anechoic tank usually used for ships’ sonar calibration. The name Wolfset is inspired by the Seawolf submarine class, renowned for its advanced sound source detection and classification capabilities. Using an anechoic tank, we can obtain a high-quality dataset representing acoustic sources without undesired external perturbations. In many operating conditions, several outboard motors and an electric motor from a basic remotely controlled ship model were used as sound sources, usually called targets. Then, external transients and noise sources were added to approximate the dataset to the sounds present in real-world conditions. This dataset uses a systematic approach to demonstrate the diversity and accuracy needed for effective algorithm development.

## Background & Summary

Data, information, and knowledge are essential concepts in our daily lives^1^. Data is critical since its processing and analysis can provide crucial information and knowledge at a final stage, which can bring advantages depending on the field of action. Nowadays, data analysis is mainly performed using Machine Learning (ML) and Artificial Intelligence (AI) and can be applied to supply chain management^2^, power electronics^3^, healthcare^4^, engineering design^5^, underwater acoustic^6^, among many others fields of science and actuation. With the expansion of data science^7^, more data is continuously becoming available, introducing new problems such as dataset standardization^8^ that must be appropriately addressed. Data standardization involves converting data into a common format to facilitate third-party analysis and processing, increasing interoperability^9,10^.

Sound waves travel by vibrating particles, and their speed and range depend on the elasticity of the propagation environment^11^. Underwater acoustics is the branch of science that studies how sound waves propagate in the dense and elastic water environment and how they interact with it^12,13^. In general, oceans and rivers contain many sources of noise that can be classified as either anthropogenic^14^ or natural^15^. Anthropogenic sounds originate from sources such as ships and coastal zone operations, while natural sounds come from animals, rain, sea movement, and other sources. An anthropogenic dataset capable of being used to help develop new solutions and algorithms during research and development in underwater acoustics is essential since its acquisition is usually expensive and time-consuming.

In all navies worldwide, submariners share the same motto: *There are only two types of ships, submarines and targets*. To obtain operational advantage and be able to call others *targets*, it is essential to capture and classify underwater sound sources correctly, since it is necessary for many tasks, including the task of standard surveillance^13^. Coastal monitoring systems typically rely on a Vessel Traffic System (VTS) equipped with radar, electro-optical capabilities, and the ability to receive data from the Automatic Identification System (AIS)^16^. However, heavy maritime traffic areas pose a challenge as small vessels and submarines tend to evade surveillance^17^, which is a significant concern, especially with the increasing use of submarines due to armed conflicts in Eastern Europe^18^.

**Fig. 1:**
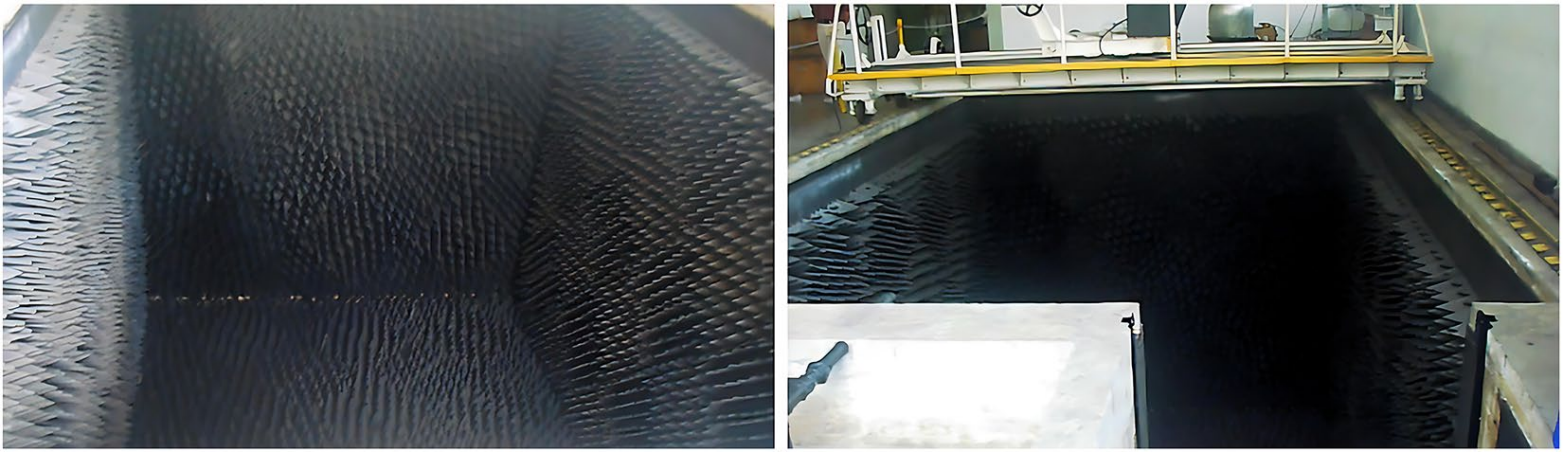
The empty anechoic tank features absorbent plates shaped like spikes made of cork agglomerates and rubber to minimize sound reflections.

Some currently underwater acoustic datasets^19,20^ contain some data acquired directly from the sea, but that uncontrolled environment is subject to undesired noise and perturbations. Acquiring acoustic data in controlled and well-defined scenarios, such as anechoic tanks containing absorbent plates made of cork agglomerates and rubber to minimize sound reflections for ships’ sonar calibration, can be expensive (Fig. 1). It is essential to ensure that the data gathered in those scenarios can be successfully reused. These ideal test conditions ensure a high-quality dataset representing the sound sources without undesired external perturbations. The hydrophonic effects are an essential aspect of underwater acoustics research, and their collection and analysis can be challenging. To aid this endeavor, we have gathered a dataset of hydrophonic effects, non-classified for security purposes, named Wolfset^21^. Usually, this kind of dataset is classified and gathered in the sea, where we have several undesired noise sources. The name Wolfset is inspired by the Seawolf submarine class, renowned for its advanced sound source detection and classification capabilities. This dataset can benefit various research projects and develop techniques that may automatically identify and classify these effects since the hydrophonic effects present in the dataset closely resemble real effects observed at sea. Using real data and not synthetic data also increases the dataset value since we can develop algorithms that perform well in real-world applications. Its size and content can also guarantee statistical significance and the demanded diversity. We have acquired and pre-processed a quality dataset representing outboard motors and a basic remotely controlled ship model as targets, that can be easily used to perform, e.g., classification^22–24^. Adding external transient effects and noise to the dataset increases its proximity to reality, which is expected during algorithm design and analysis. Since we have samples with and without adding manual noise, it is possible to add different types of noise using preprocessing to better mimic the desired application.

**Fig. 2:**
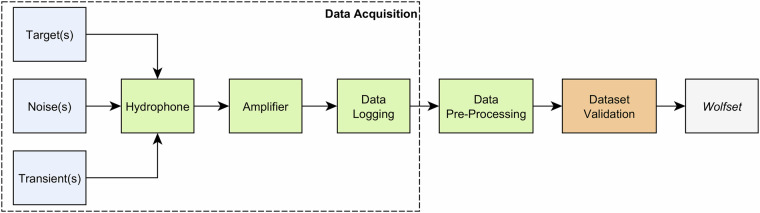
Simplified diagram of the dataset creation scheme.

The final dataset^21^ consists of about 1.5 gigabytes of data, encompassing 5 hours of recordings in WAVEform (WAVE) audio format. A simplified diagram describing all the steps involved in the dataset creation is illustrated in Fig. 2. The data acquisition was performed using a Bruel & Kjaer type 8104 hydrophone^25^ followed by a two-stage adjustable gain signal amplifier Bruel & Kjaer 2636^26^. The *Data Logging* was performed using a simple computer without any specific computational requirements, and the *Data Pre-Processing* stage only entailed adjusting the file duration and content to ensure the dataset uniformity and consistency. A technical data validation was performed for *Dataset Validation*, to ensure the quality of the final dataset.

## Methods

The accuracy and reusability of the final dataset depend heavily on the acquisition conditions. It is crucial to consider the conditions of the acquisition environment, including the anechoic tank, the used hydrophone and signal amplifier, and the accuracy of the data acquisition process. All hardware and data acquisition processes should be carefully selected and executed to ensure the usefulness and accuracy of the dataset. Since we are dealing with a costly and time-consuming acquisition process, we must ensure the reuse capability of the acquired dataset.

### Anechoic Tank

**Fig. 3:**
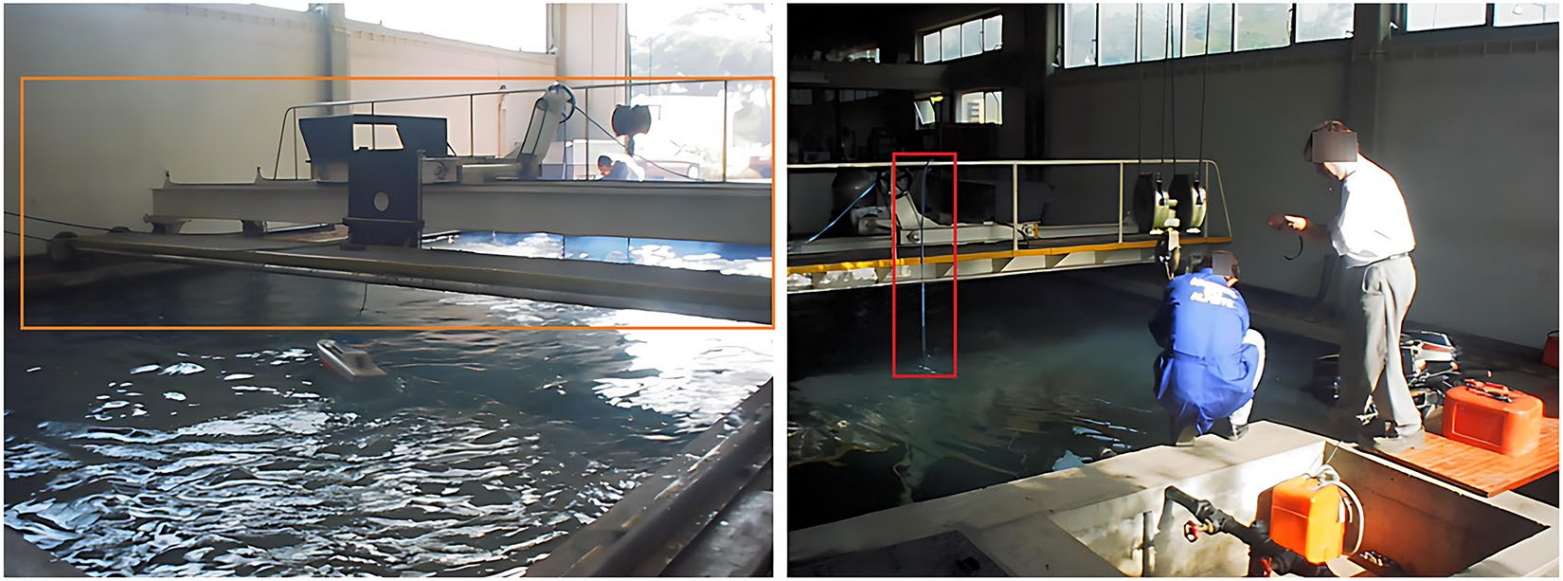
The overall appearance of the tank is illustrated here with two movable bridges shown in orange (*left*) and the data acquisition system that suspends the hydrophone from one of the bridges, illustrated in red (*right*).

The anechoic tank was constructed in 1976 at the Lisbon Naval Base and is primarily used for calibrating ships’ sonar, which is regularly used today by the Portuguese Navy to calibrate their underwater sensors and systems. Its surface is covered with absorbent plates made of cork agglomerates and rubber, which help to reduce sound reflections. In addition, floating plates can be added or removed from the tank surface. The plates guarantee a density of approximately *ρ* ≅ 0.8 g/cm^3^. The tank is 8 meters long, 5 meters wide, and 5 meters deep. Its design includes a small auxiliary tank in one corner, separated from the main tank by a floodgate. Two movable bridges cross the tank from one end to the other, allowing better access to all the tank areas, as shown in Fig. 3.

Since the anechoic tank is used periodically, it undergoes periodic and corrective maintenance throughout the year to ensure precision in the performed tests, sensors, and systems calibration. The absorbent plates are periodically substituted when they lose their properties, providing the perfect test conditions to guarantee the accuracy of the gathered acoustic dataset^21^.

### Target, Noise & Transient Sound Sources

**Fig. 4:**
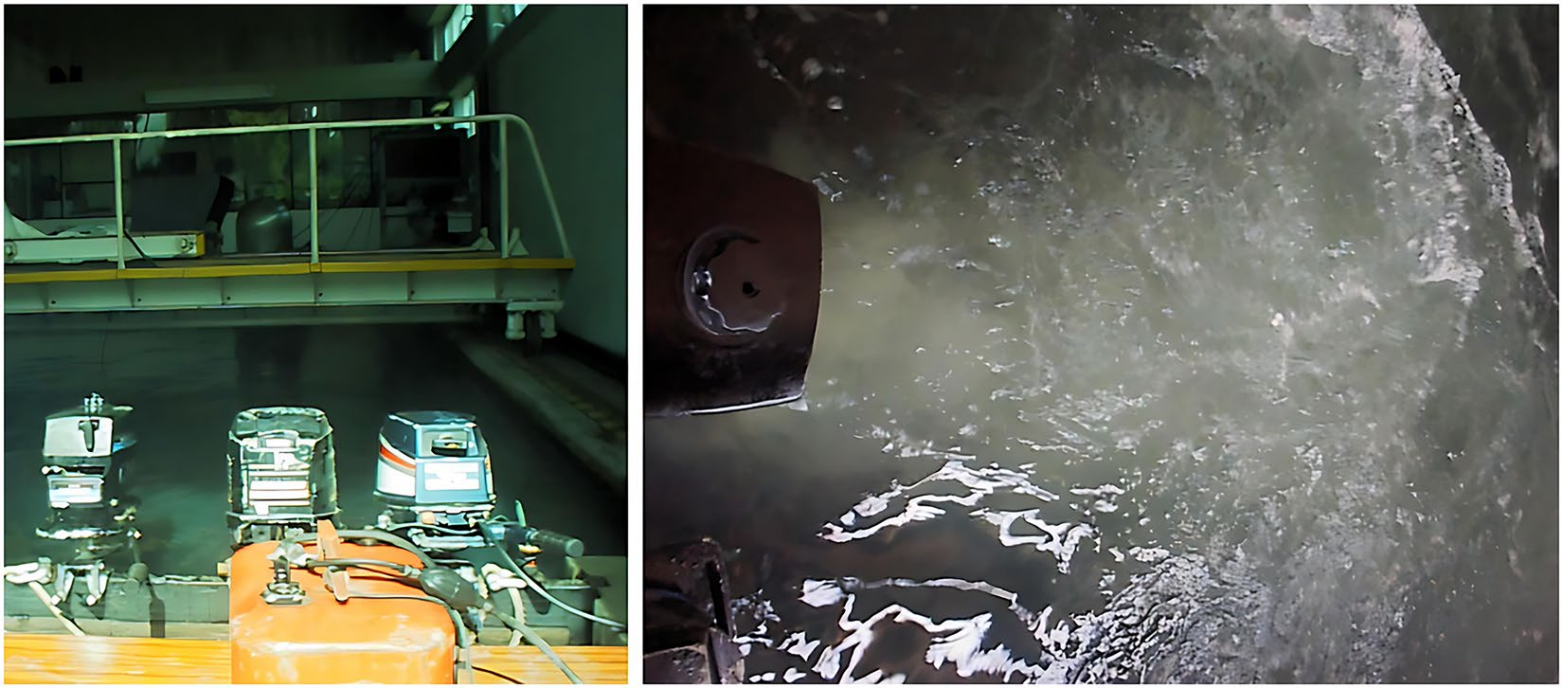
Illustration of three of the utilized motors (*left*) and the bubbles generated across the water surface (*right*).

As target sound sources, outboard motors were attached to the floodgate gate during the data acquisition, as shown in Fig. 4. An electric motor from a basic remotely controlled ship model was also used to improve the dataset diversity.

**Fig. 5:**
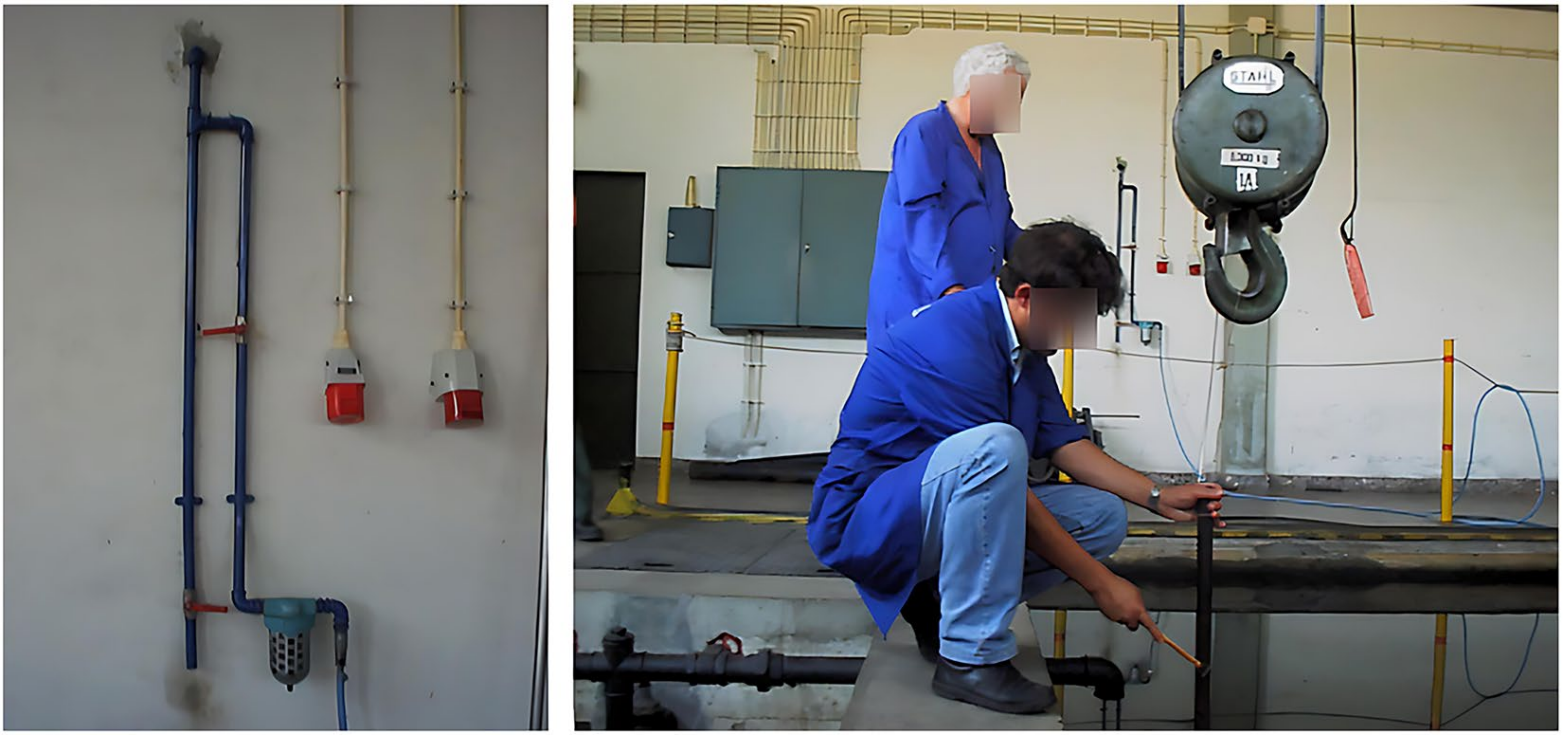
The water inlet filter (*left*) and the crane used to transport cargo along the tank (*right*).

During the dataset acquisition process, one of the two existing movable bridges (Fig. 3) was used to suspend the hydrophone, and the other was used to suspend the compressed air hoses. These compressed air hoses were turned on when needed to generate bubbles as noise, guaranteeing that the acquired dataset^21^ would possess sounds that are as close to the expected reality conditions as possible. A water pumping system with a discharge located approximately 15 cm above the surface was also used to generate noise in the recordings, allowing for the inclusion of background noise. Some transients were also created using, e.g, metallic bars or shots from an air rifle. To perform the placement of the motors and all the necessary materials, a crane that supports up to 5 tons was used, as shown in Fig. 5.

### Hydrophone & Signal Amplifier

**Fig. 6:**
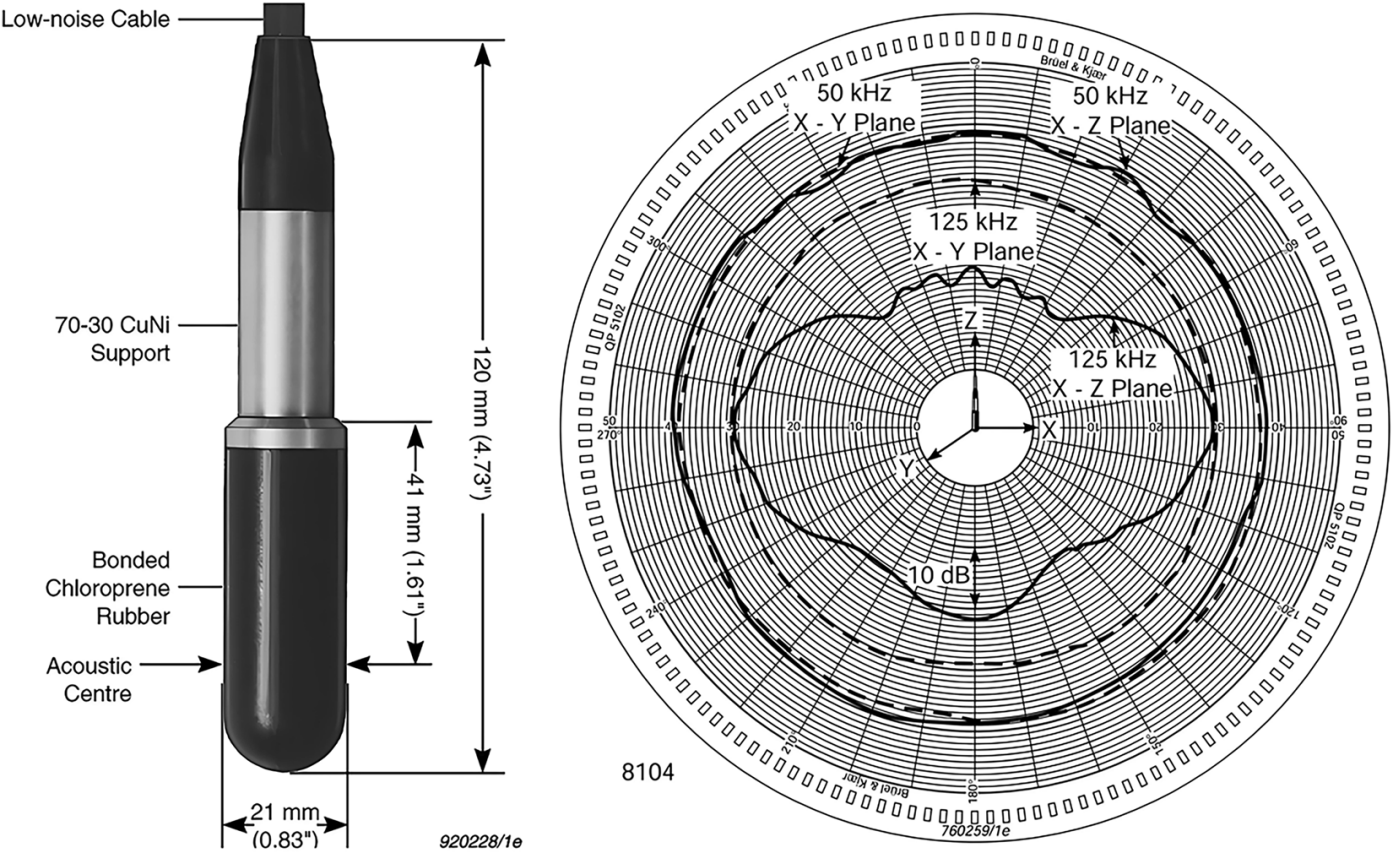
Hydrophone Bruel & Kjaer type 8104 (*left*) and its typical directivity pattern (*right*)^37^.

A Bruel & Kjaer type 8104 hydrophone^25^, commonly used in acoustic data applications^27–29^, was employed during the dataset acquisition, located in the middle of the tank: 2.5 meters deep, 2.5 meters from the lateral walls, and 4 meters from both ends of the tank. This hydrophone is a passive omnidirectional device, 12 cm in length and 2 cm in diameter, weighing approximately 1.3 kg, as illustrated in Fig. 6*left*. The typical directivity pattern for this hydrophone model is shown in Fig. 6*right*, illustrating how the transfer function varies with the frequency and the location of the sound source relative to the hydrophone. This hydrophone can capture signals ranging from 0.1 Hz to 200 kHz and features a constant directivity pattern up to 20 kHz, as confirmed during calibration before dataset acquisition. In frequency, the transfer function of the transducer is almost flat up to the sampling frequency we used.

During the data acquisition, a two-stage adjustable gain signal amplifier Bruel & Kjaer 2636^26^ was used, and the gain used on each stage in *dB* is discriminated in the dataset annotation file^21^, as described in the *Data Records* section. During all the performed tests, the amplifier was equipped with programmable filters that served as low-pass filters with a selected cutoff frequency of 22.4 kHz.

### Data Logging

Following the amplifier, we used a high-quality Hewlett-Packard (HP) oscilloscope and a spectral analyzer to monitor the signals being measured. This setup allows us to fine-tune the gain manually to use most of the dynamic range for a good representation of the important (stationary) signals. But, since the gain is fixed, to mimic the expected operational conditions, there is sometimes saturation of the signal with loud transient noises.

We received the audio signal using a 16-bit sound card that is currently used for standard ship sonar calibration operations conducted at the anechoic tank. Laboratory tests revealed that the card’s transfer function was almost flat from 50 Hz to 20 kHz and could sample and quantize signals as low as 1 Hz. Since the same sound card was used for all recordings, all very low-frequency signals were consistently affected. All recordings were made at a 44.1 kHz sampling rate in mono channel mode.

### Data Acquisition

**Fig. 7:**
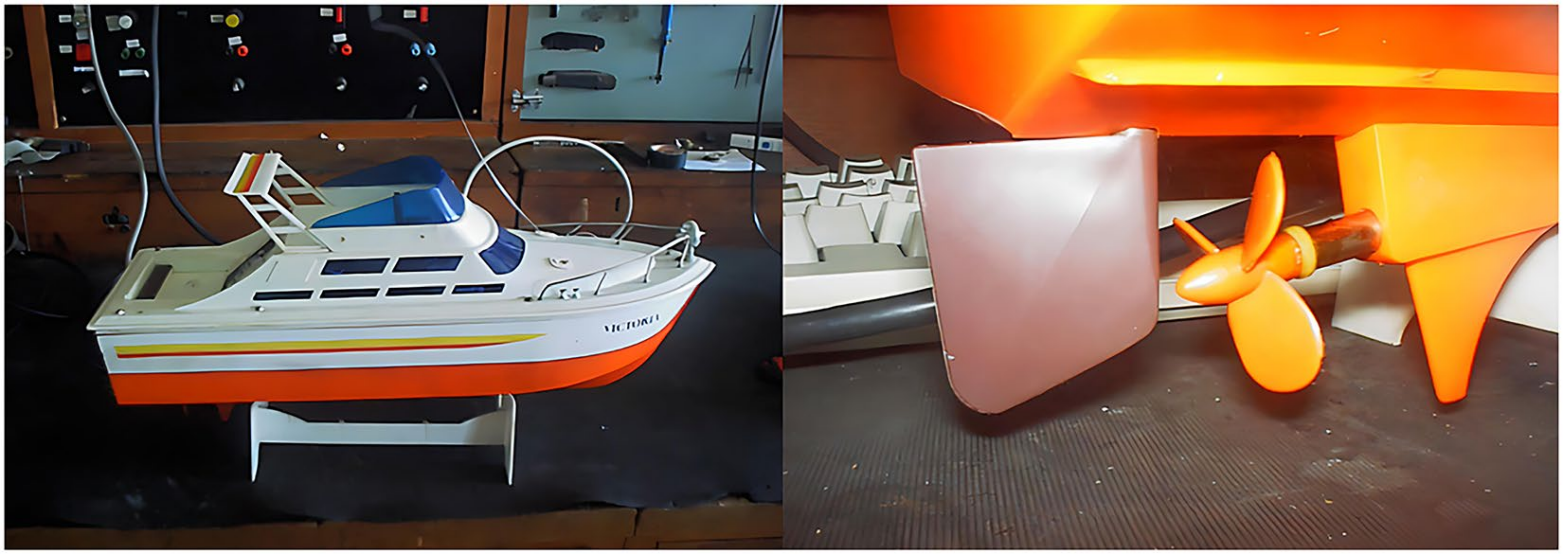
Used electric model (*left*) and the model 3-blade propeller (*right*).

**Fig. 8:**
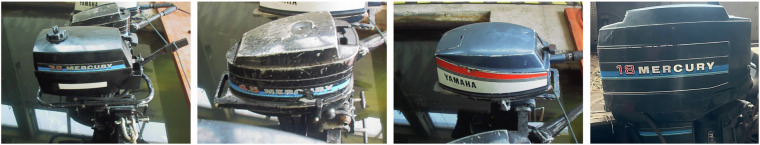
Outboard *Mercury* motors: 3.6 horsepower (*left*), 4.5 horsepower (*center left*), 8 horsepower (*center right*), and 18 horsepower (*right*).

During the data acquisition, we used as targets an electric motor from a basic remotely controlled ship model (Fig. 7) and four different outboard *Mercury* motors (Fig. 8). The outboard motors used were four *Mercury* models^30^ with the following characteristics: (i) a 4.5 horsepower motor with a right pitch propeller having three blades, (ii) an 18 horsepower motor with a right pitch propeller having three blades, (iii) an eight horsepower motor with a right pitch propeller having three blades, and (iv) a 3.6 horsepower motor with a right pitch propeller having three rubber blades.

**Fig. 9:**
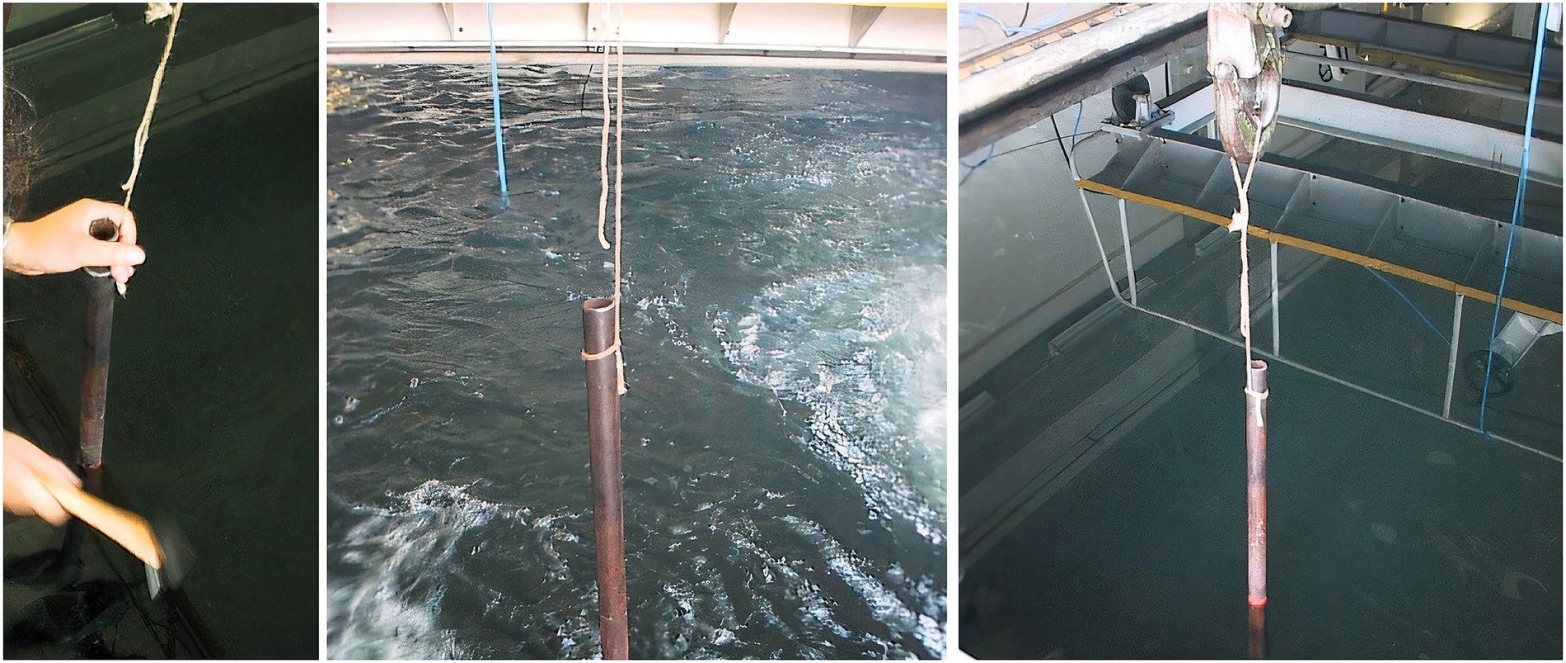
Metallic bars used to generate the transients present in the dataset.

**Fig. 10:**
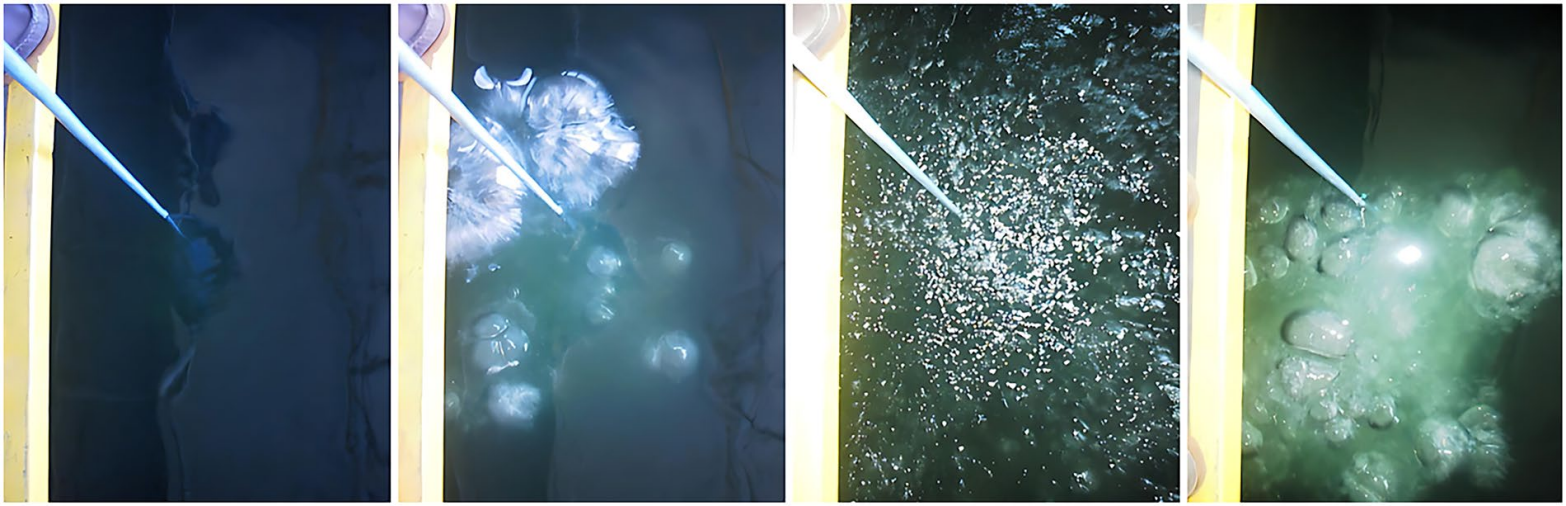
Compressed air bubbles creation using compressed air hoses to generate noise in the dataset.

**Fig. 11:**
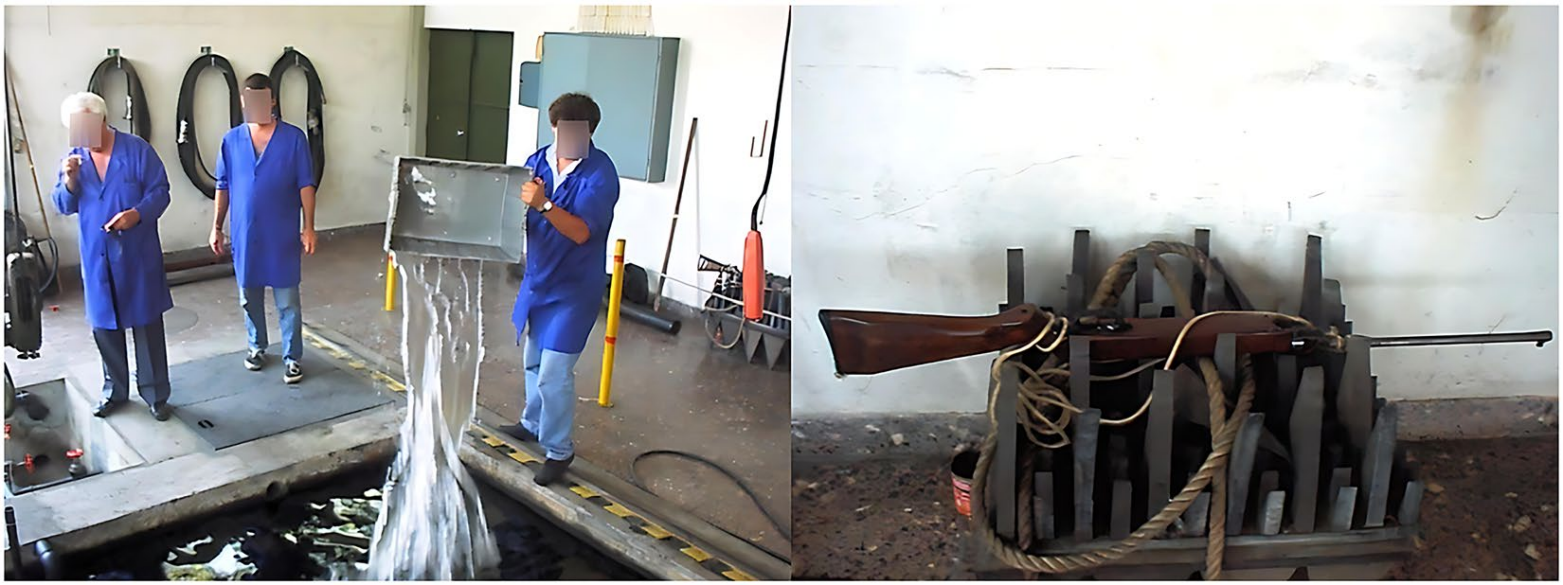
The water bucket (*left*) and air rifle (*right*) used to generate transients in the dataset.

To create some transients and background noise, we have used metallic bars (Fig. 9), compressed air (Fig. 10), a water bucket (Fig. 11*left*), and shots from an air rifle (Fig. 11*right*). All the sound sources and respective added noise and transients will be appropriately described in the *Data Records* section.

The targets were chosen based on their prevalence in small vessels, including four combustion motors and one electric motor, to ensure variability in acoustic signatures, which is crucial for developing and testing algorithms. Additionally, the manually added noise and transients provide authentic sound sources reflective of typical operational interferences in real-world underwater systems, rather than relying solely on simulated data. This approach ensures diverse acoustic profiles, featuring distinct frequency bands and transient characteristics. The quality of the dataset, derived from all the adopted acquisition procedures, ensures its suitability for reuse in future studies.

## Data Records

The dataset^21^ comprises approximately 1.5 gigabytes and includes 168 WAVE audio format files, totaling about 5 hours of audio recordings. It also contains two annotation files named *Wolfset_Index.xlsx* and *Wolfset_Index.csv*, each providing a summary of the contents of the respective WAVE files. The file names indicate the content of the corresponding recordings according to the following template *XxxxxxTttNnn.WAV*, with respective code given by:

- *X* - It can take two values: (i) *A* - which will correspond to a regular recording, and (ii) *E* - which will correspond to a recording where some error or occurrence happened during the test, which will be described in the annotation file;
- *xxxxx* - Corresponds to the intensity code of each target. The first digit refers to motor 1 (4.5 horsepower), the second to motor 2 (18 horsepower), the third to motor 3 (8 horsepower), the fourth to motor 4 (3.6 horsepower), and the fifth to motor 5 (electric motor model). For motors 1 to 4, the annotation can take one of the following values:
  - Value *0* - Absent (or disconnected);
  - Value *1* - Idle disengaged;
  - Value *2* - Idle engaged forward;
  - Value *3* - Slow forward;
  - Value *4* - Medium forward;
  - Value *5* - Varying (15 seconds out of gear with small accelerations, then accelerations with the propeller engaged).
  For motor 5, since it is a remotely controlled electric ship model, the annotation can take one of the following values:
  - Value *0* - Absent (or disconnected);
  - Value *1* - Slow forward;
  - Value *2* - Fast forward;
  - Value *3* - Slow reverse;
  - Value *4* - Fast reverse.
- *T* - It is the initial for *Transient*;
- *tt* - The transient codes form a number that is the sum of the weights of the different effects, according to:
  - Decimal *0* - Without any transients;
  - Decimal *1* - Compressed air in discharges;
  - Decimal *2* - Water Bucket discharge;
  - Decimal *4* - Hitting the metallic tube with a mallet;
  - Decimal *8* - Hitting the metallic tube with an hammer;
  - Decimal *16* - Air rifle shot.
- *N* - It is the initial for *Noise*;
- *nn* - The noise codes form a number that is the sum of the weights of the different effects, according to:
  - Decimal *0* - Without noise;
  - Decimal *1* - Compressed air bubbling very intensely (tap fully open);
  - Decimal *2* - Compressed air bubbling at low intensity (tap open at 1/4);
  - Decimal *4* - Water hose with low flow (tap open with just one turn);
  - Decimal *8* - Water hose with a lot of flow (tap fully open).
- **General Notes**:
  1. If there are multiple recordings under the same conditions, they will have an additional suffix denoted by *xNN*, where *NN* represents the recording number;
  2. For instance, if we have a recording labeled *A01020T00N05.WAV*, we have:
    *A01020* - Recording includes motor 2 at *Idle disengaged* and motor 4 at *Idle engaged forward*;
    *T00* - The recording does not present any transient fluctuations;
    *N05* - Recording including noise from *Compressed air bubbling very intensely* and *Water hose with low flow*.

A summary of the dataset filename structure and annotation codes is presented in Table 1. The dataset^21^ includes a diverse collection of recordings categorized by noise, transients, targets, and combinations. Table 2 details the subset containing only noise, encompassing four distinct noise types and one combined scenario, resulting in 34.13 minutes of dedicated noise recordings.

**Table 1:** Summary of the dataset filename structure and annotation codes.

Code Segment	Meaning	Description / Values
*X*	Recording Type	**A**: Regular recording **E**: Recording with an event or anomaly (described in annotation file)
*xxxxx*	Motor Activity Code	5-digit code: Each digit corresponds to one motor 1st-4th digits: Motors 1-4 (Combustion) 5th digit: Electric motor (ship model) **Motors 1-4:** 0 = Absent, 1 = Idle disengaged, 2 = Idle engaged forward, 3 = Slow forward, 4 = Medium forward, 5 = Varying **Motor 5:** 0 = Absent, 1 = Slow forward, 2 = Fast forward, 3 = Slow reverse, 4 = Fast reverse
*T*	Transient Section	Always the letter *T*, marking the beginning of the transient code
*tt*	Transient Code	Sum of weights representing combined transient events: 0 = None, 1 = Air discharge, 2 = Water bucket, 4 = Tube hit (mallet), 8 = Tube hit (hammer), 16 = Air rifle
*N*	Noise Section	Always the letter *N*, marking the beginning of the noise code
*nn*	Noise Code	Sum of weights representing combined noise sources: 0 = None, 1 = Air bubbling (high), 2 = Air bubbling (low), 4 = Hose (low flow), 8 = Hose (high flow)
*xNN*	Recording Number	Optional suffix for repeated setups (e.g., *x01*, *x02*)
		Used when multiple recordings exist with the same configuration

**Table 2:** Summary of the dataset recordings containing only noise.

Code	Noise	Number & Duration
*N01*	Compressed air bubbling very intensely	2 × 1 min & 1 × 21.1 min
*N02*	Compressed air bubbling at low intensity	2 × 1 min
*N04*	Water hose with low flow	2 × 1 min
*N08*	Water hose with a lot of flow	2 × 1 min
*N09*	Combination of *N01* and *N08*	1 × 5.03 min

Another essential component of the dataset^21^ is the inclusion of transient acoustic events, which enhance its realism by reflecting common occurrences in real underwater environments. Table 3 summarizes the recordings containing only transients, comprising five distinct types of sounds. Among these, the air rifle shot was recorded 32 times in 10-second segments. In contrast, the remaining four transient types–water bucket discharges and impacts with a mallet or hammer–were each recorded over five-minute sessions with repeated events, offering a diverse set of non-stationary acoustic signatures.

**Table 3:** Summary of the dataset recordings containing only transients.

Code	Transient	Number & Duration
*T01*	Compressed air discharges	1 × 5 min
*T02*	Water bucket discharge	1 × 5 min
*T04*	Striking a metallic tube with a mallet	1 × 5 min
*T08*	Hitting a metallic tube with a hammer	1 × 5 min
*T16*	Air rifle shots	32 × 10 s

As stated, four combustion motors were employed to simulate the acoustic signatures typically associated with small vessels. The dataset includes recordings for motors 1 through 4, each captured under five defined intensity levels, initially without any added noise or transient interference. To further enrich the dataset, additional recordings were conducted with each motor operating at the *Idle engaged forward* and *Medium forward* states, incorporating controlled noise and transient events–these transients were repeated six times to simulate realistic disturbances. A detailed summary of all recordings performed with these motors is presented in Table 4, with total durations of 54 minutes for motor 1, 52 minutes for motor 2, 57 minutes for motor 3, and 45.8 minutes for motor 4.

**Table 4:** Summary of the recordings performed with motors 1 to 4 under various operating conditions.

Condition	Motor 1	Motor 2	Motor 3	Motor 4
Idle disengaged	5 × 1 min	2 × 5 min	2 × 5 min	1 × 5.8 min
Idle engaged forward	1 × 5 min	1 × 5 min	2 × 5 min	1 × 6 min
Slow forward	5 × 1 min	1 × 5 min	1 × 5 min	1 × 7 min
Medium forward	5 × 1 min	1 × 5 min	1 × 5 min	1 × 5 min
Varying	2 × 1 min	2 × 1 min	2 × 1 min	2 × 1 min
Idle engaged forward + *N01*	4 × 1 min	1 × 5 min	1 × 5 min	1 × 2 min & 1 × 5 min
Idle engaged forward + *N02*	4 × 1 min	–	–	–
Idle engaged forward + *N08*	4 × 1 min	1 × 5 min	–	–
Idle engaged forward + *N04*	4 × 1 min	–	–	1 × 5 min
Medium forward + *N01*	1 × 5 min	1 × 5 min	1 × 5 min	1 × 5 min
Medium forward + *N08*	1 × 5 min	1 × 5 min	–	–
Medium forward + *N04*	–	–	1 × 5 min	1 × 5 min
Idle engaged forward + *T01*	1 × 1 min	1 × 1 min	1 × 1 min	1 × 1 min
Idle engaged forward + *T02*	1 × 1 min	1 × 1 min	1 × 1 min	1 × 1 min
Idle engaged forward + *T04*	1 × 1 min	1 × 1 min	1 × 1 min	1 × 1 min
Idle engaged forward + *T08*	1 × 1 min	1 × 1 min	1 × 1 min	1 × 1 min
Idle engaged forward + *T16*	2 × 1 min	1 × 1 min	1 × 1 min	1 × 1 min

Motor 5, an electric unit representative of low-noise propulsion systems used in small-scale platforms, was recorded at its four predefined intensity levels. To capture specific behaviors, additional one-minute recordings were included featuring rudder-induced effects–one at *Slow forward* and two at *Fast forward*–resulting in a total of seven minutes of data, as detailed in Table 5. These comprehensive recordings, across varying motor types and conditions, contribute to the dataset’s robustness and applicability for underwater acoustic analysis and machine learning applications.

**Table 5:** Summary of the recordings performed with motor 5 - Electric.

Condition	Number & Duration
Slow forward	1 × 1 min
Fast forward	1 × 1 min
Slow reverse	1 × 1 min
Fast reverse	1 × 1 min
Slow forward (*with rudder beats*)	1 × 1 min
Fast forward (*with rudder beats*)	2 × 1 min

These recordings cover various operational conditions and are further enriched by including controlled noise and transient events, as outlined in the corresponding tables. This combination enhances the dataset’s diversity and realism, making it well-suited for research in underwater acoustics and advanced signal processing techniques. A subset of the data also includes recordings where all motors operated simultaneously at the *Idle engaged forward* intensity, as shown in Table 6.

**Table 6:** Summary of the recordings performed combining the motors.

Motor Combination	Number & Duration
Motor 1 + Motor 2	1 × 1 min
Motor 1 + Motor 3	1 × 1 min
Motor 1 + Motor 4	1 × 1 min
Motor 1 + Motor 5	1 × 1 min
Motor 2 + Motor 3	1 × 1 min
Motor 2 + Motor 4	1 × 1 min
Motor 2 + Motor 5	1 × 1 min
Motor 3 + Motor 4	1 × 1 min
Motor 3 + Motor 5	1 × 1 min
Motor 4 + Motor 5	1 × 1 min
Motor 1 + Motor 3 + Motor 4	1 × 1 min (Motor 3 stopped at 20 seconds)

The dataset was developed under highly controlled conditions using an anechoic tank and a calibrated hydrophone, ensuring high-fidelity recordings suitable for precise acoustic analysis. It also features recordings of combined motor operations to simulate complex real-world scenarios. This includes all ten possible pairings of motors 1 through 5 and one scenario with three motors (1, 3, and 4) operating simultaneously. In the latter, motor 3 stopped running at the 20-second mark of the one-minute recording. For all combined recordings, motors 1 to 4 were generally set to *Idle engaged forward*, and motor 5 to *Fast forward*. Two exceptions were made: in the pairing of motors 2 and 4, motor 2 operated at *Idle engaged forward* while motor 4 ran at *Medium forward*, and in the combination of motors 4 and 5, motor 4 was set to *Idle disengaged* and motor 5 to *Slow forward*. These combined recordings do not introduce noise or transients, allowing for clean analysis of motor interaction sounds.

## Technical Validation

**Fig. 12:**
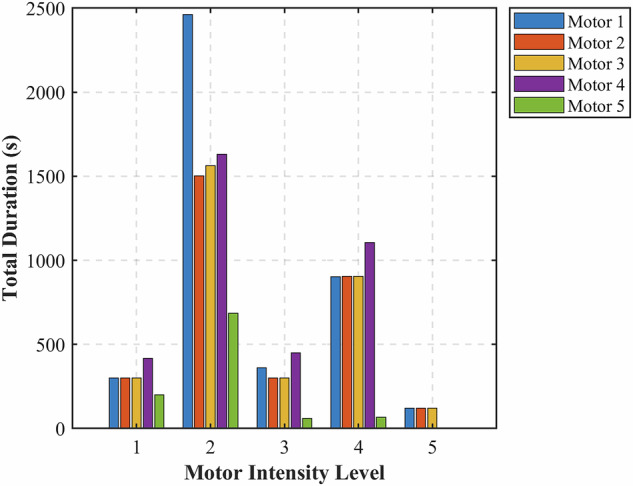
The comparison of file duration in seconds, accounting for intensity levels across motors.

This section further validates the dataset’s quality by analyzing only the active motor states. Fig. 12 illustrates each motor’s total duration in seconds across intensity levels 1 to 5, excluding the inactive state. The data reveals that intensity level 2 (*idle engaged forward*) dominates across most motors, since this operational mode was the primary focus during testing, as it reflects a common propulsion state in small vessels loitering in a certain area. Intensity levels 3 (*slow forward*) and 4 (*medium forward*) are also well represented, providing valuable examples of transitional and moderate thrust behavior. Including all defined active intensities contributes to a more comprehensive dataset, supporting robust development and validation of underwater acoustic target recognition models. Moreover, the differences in duration distributions among motors at the same intensity indicate a diverse and realistic collection of acoustic conditions, likely achieved through purposeful test variation and scenario-based data acquisition.

**Fig. 13:**
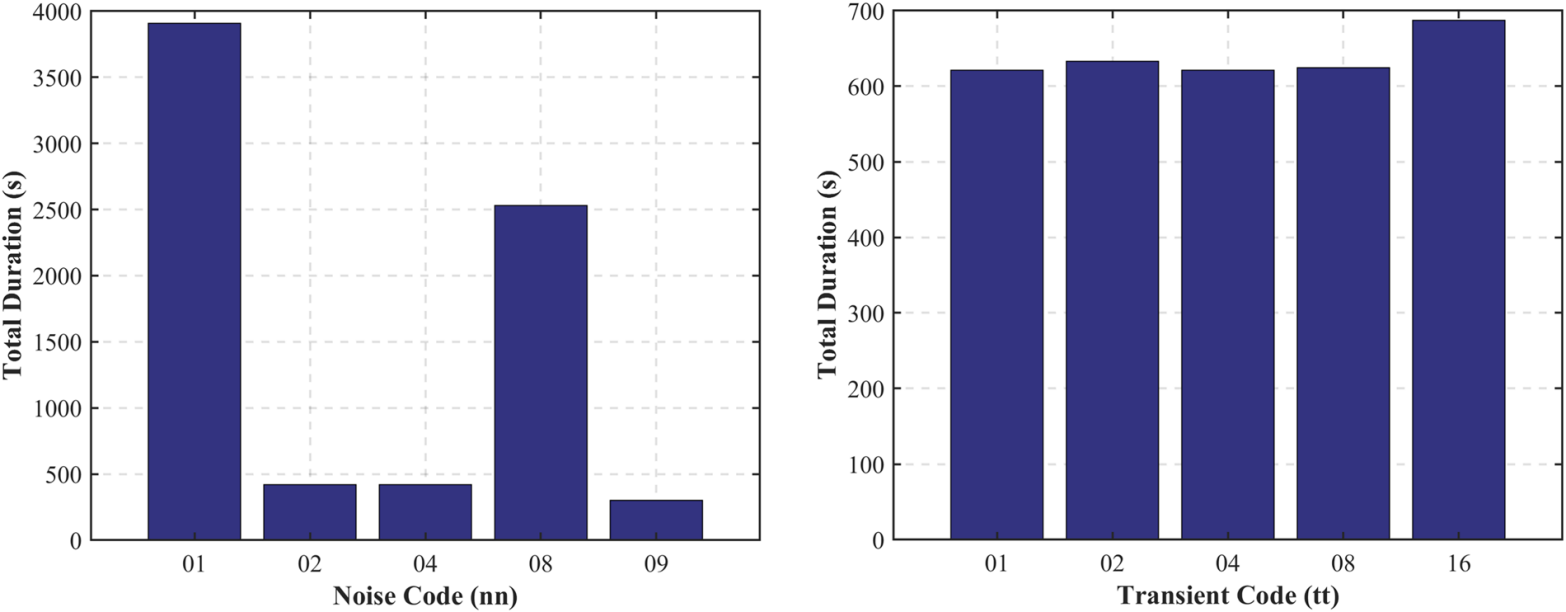
The comparison of file duration according to its code: Noise (*left*) and Transient (*right*).

Additionally, the total duration of the dataset’s transient and noise recordings^21^–excluding code 00–is illustrated in Fig. 13. The transient code chart reveals a well-balanced distribution across all transient types, including air discharges, water bucket drops, metallic impacts, and air rifle shots, each contributing similarly to the overall dataset duration. This uniformity indicates deliberate test design to cover various impulsive acoustic events adequately. In contrast, the noise code distribution displays a more heterogeneous profile, with codes 01 (*intense air bubbling*) and 08 (*high-flow water hose*) representing most of the noise-related duration. This suggests a particular emphasis on simulating turbulent or high-energy background conditions. These figures confirm that, although transients and noise comprise a smaller portion of the dataset, their inclusion is purposeful and sufficiently diverse to enhance its applicability in testing acoustic models under complex, real-world scenarios involving sudden events and dynamic background noise.

To complement the validation of the data, visualizing the Fast Fourier Transform (FFT)^31^ and the Spectrogram^32^ can assist in analyzing and drawing conclusions about the dataset’s quality and content by identifying dominant frequencies, assessing noise levels, detecting anomalies, and understanding the temporal evolution of frequency components, which are essential for evaluating the dataset’s consistency and integrity.

**Fig. 14:**
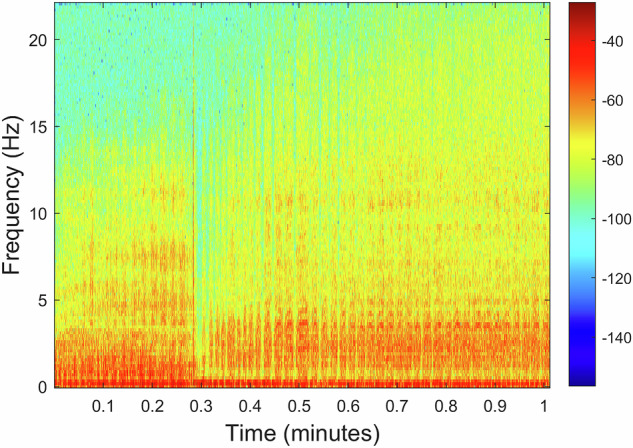
Recording *A00500T00N00*×*01.WAV* spectrogram: Motor 3 with varying speeds without any additional noise source.

The recording *A00500T00N00*×*01.WAV* includes motor 3 operating at varying speeds without any additional noise source, whose spectrogram is illustrated in Fig. 14. Low-frequency bands showcase the motor’s primary rotational speed and its harmonics. Frequency shifts represent variations in speed, with brighter (red) regions signifying a high presence of specific frequencies in the analyzed recording.

**Fig. 15:**
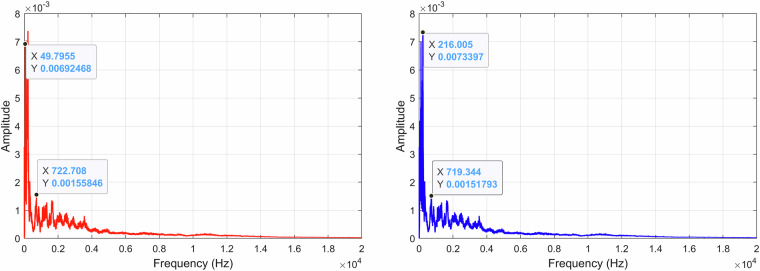
Recording *A00500T00N00*×*01.WAV* normalized FFT with the original recording (*left*) and the filtered recording (*right*).

**Fig. 16:**
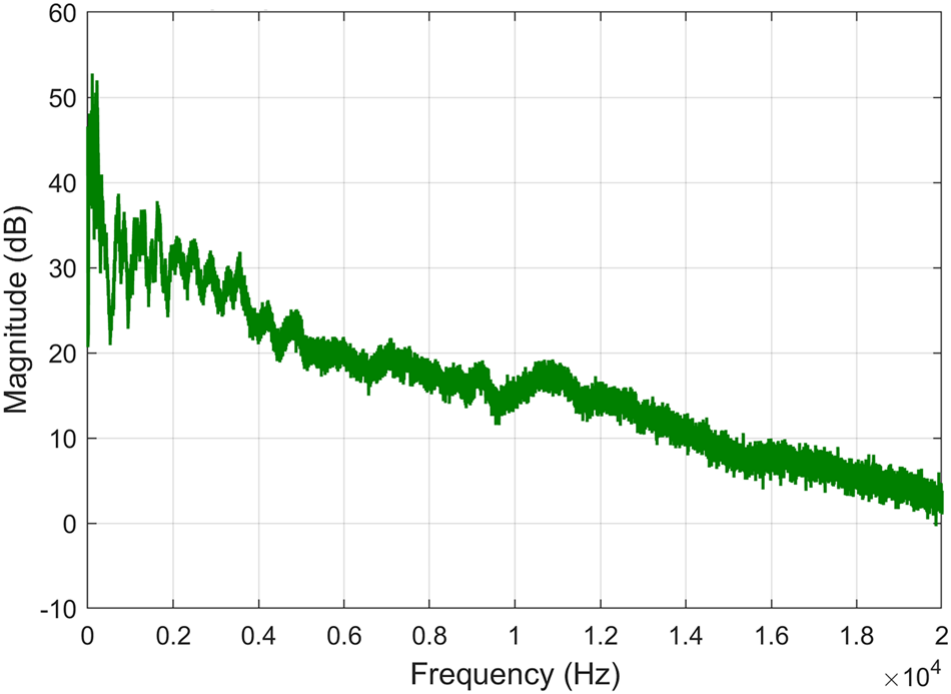
FFT of the *A00500T00N00*×*01.WAV* recording using a decibel scale.

When analyzing the recording *A00500T00N00*×*01.WAV* by viewing the FFT of all the signal, it is possible to see the existence of an approximately 50 Hz component originating from the electrical supply network, as illustrated in Fig. 15. By filtering the 50 Hz component and its harmonics up to 200 Hz with a standard second-order Infinite Impulse Response (IIR) notch filter, we eliminate this undesired signal component without compromising our spectrum. The FFT was computed using a Hanning (actually Von Hann^33^) window of *N* = 65, 536 samples, yielding a frequency resolution of approximately 0.67 Hz, and the result is shown as the average over 40 time blocks. A FFT representation of the *A00500T00N00*×*01.WAV* recording using a decibel scale is shown in Fig. 16.

**Fig. 17:**
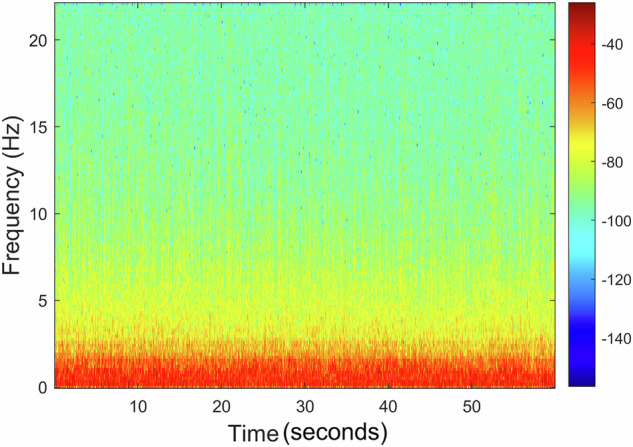
Recording *A00000T00N01*×*01.WAV* spectrogram: Presence of noise caused by compressed air bubbling intensely without any target.

The recording *A00000T00N01*×*01.WAV* includes only the presence of noise caused by compressed air bubbling intensely without any target, with the spectrogram illustrated in Fig. 17. A comparison of the obtained spectrogram with the one shown in Fig. 14 reveals that this new spectrogram reflects a stable signal characterized by sustained low-frequency energy over an extended time frame. In contrast, the earlier spectrogram represents considerable fluctuations in frequency content over a shorter duration, illustrating the dynamic changes in the motor speed.

**Fig. 18:**
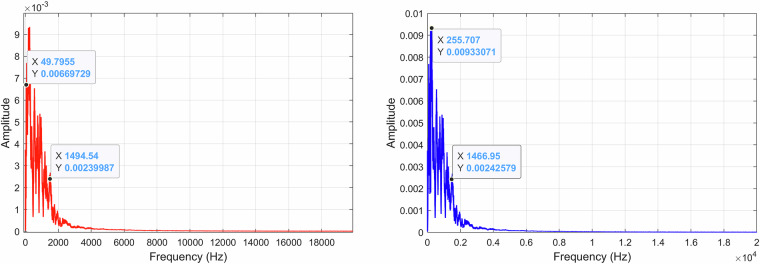
Recording *A00000T00N01*×*01.WAV* normalized FFT with the original recording (*left*) and the filtered recording (*right*).

**Fig. 19:**
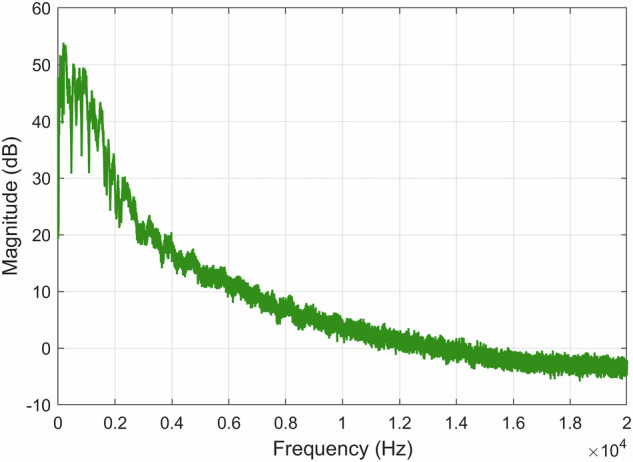
FFT of the *A00000T00N01*×*01.WAV* recording using a decibel scale.

When analyzing the recording *A00000T00N01*×*01.WAV* FFT, as illustrated in Fig. 18, it is possible to denote a higher spread of the signal frequencies across lower frequencies, mainly caused by the noise source included in the recording. Since this is an external noise source and not some noise source from the environment, its content can be used alone or, e.g., to isolate the target’s signal in other recordings or test algorithm performance in the presence of noise. Similarly, to what was done to the previous recording, the FFT was computed using also a Hanning window of *N* = 65, 536 samples. Figure 19 shows the FFT of *A00000T00N01*×*01.WAV* represented on a decibel scale.

**Fig. 20:**
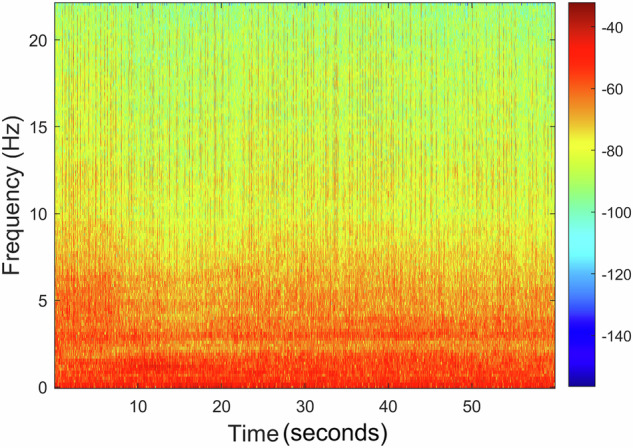
Recording *A30000T00N00*×*01.WAV* spectrogram: Motor 1 moving slowly forward without any additional noise.

The recording *A30000T00N00*×*01.WAV* includes motor 1 at a slow forward setting without any additional noise, with the spectrogram illustrated in Fig. 20. Here, we observe the presence of some lower frequencies and a spread into higher frequencies, although they remain very low.

**Fig. 21:**
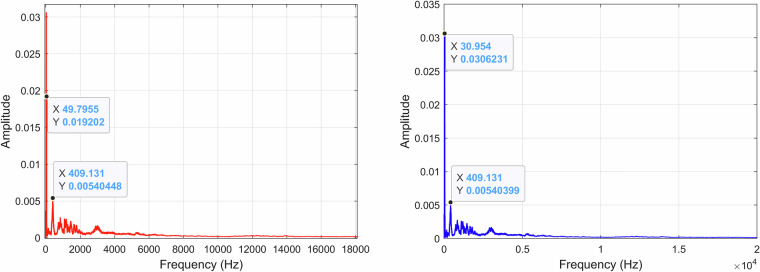
Recording *A30000T00N00*×*01.WAV* FFT with the original recording (*left*) and the filtered recording (*right*).

**Fig. 22:**
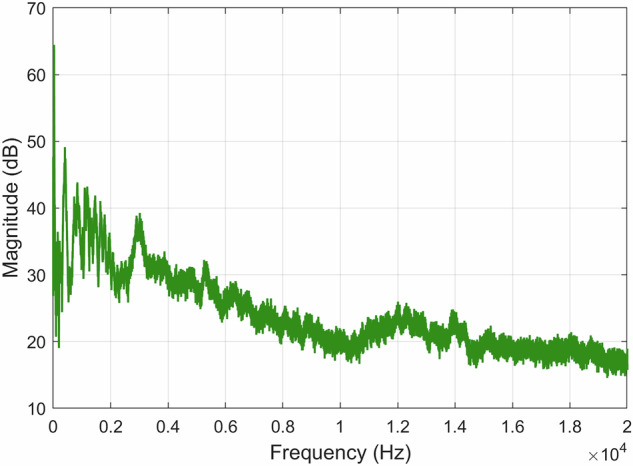
FFT of the *A30000T00N00*×*01.WAV* recording using a decibel scale.

When analyzing the recording *A00500T00N00*×*01.WAV* FFT, as illustrated in Fig. 21, it is possible to identify a component of approximately 31 Hz in the filtered recording, indicating the motor speed at slow forward. The same FFT parameters as previously mentioned were applied to *A00500T00N00*×*01.WAV* and its spectrum, using a decibel scale, is represented in Fig. 22.

The performed analysis illustrates the quality and content of the Wolfset, a dataset covering a wide range of scenarios that can aid in successful algorithm design and testing. By collecting data in ideal conditions, free from interference by other sound sources, we can ensure high-quality data that can be used confidently to classify sound sources accurately.

Data-driven approaches are increasingly essential for advancing algorithm design, especially in acoustic signal processing. However, accessing accurate, well-structured, and freely available datasets remains challenging, as data acquisition is often resource-intensive. The Wolfset dataset addresses this gap by providing high-quality underwater acoustic recordings obtained through scientifically controlled procedures. Its structure supports various research applications, allowing a reliable algorithm development and analysis source. The intentional manual inclusion of noise and transient events enhances the dataset’s realism, making it more suitable for modeling complex real-world scenarios.

## Usage Notes

This dataset^21^ can be readily used with deep learning approaches for a range of acoustic signal processing tasks, such as event monitoring^34^, unsupervised classification^35^, and event detection in noisy environments^36^. Its structured annotations and inclusion of target sources and background interferences make it especially suitable for training supervised and unsupervised models under realistic underwater conditions. Furthermore, the dataset enables experimentation in anomaly detection, acoustic scene classification, and signal enhancement. Due to its standardized naming convention and controlled acquisition process, it can also serve as a robust benchmark for comparing algorithm performance. Researchers interested in exploring advanced machine learning techniques–such as self-supervised learning, domain adaptation, or few-shot learning–will find the dataset particularly useful for method development and validation.

## Data Availability

No dedicated scripts or custom code were used to generate or process the dataset.
